# Response to the letter by Esteves et al.

**DOI:** 10.1038/s41386-018-0079-8

**Published:** 2018-04-25

**Authors:** T. S. Send, M. Gilles, V. Codd, I. A. C. Wolf, S. Bardtke, F. Streit, J. Strohmaier, J. Frank, D. Schendel, M. W. Sütterlin, M. Denniff, M. Laucht, N. J. Samani, M. Deuschle, M. Rietschel, S. H. Witt

**Affiliations:** 1 Department of Psychiatry and Psychotherapy, Central Institute of Mental Health, Medical Faculty Mannheim, University of Heidelberg, Mannheim, Germany; 2 Department of Cardiovascular Sciences, University of Leicester, Leicester, UK; 3 Department of Genetic Epidemiology in Psychiatry, Central Institute of Mental Health, Medical Faculty Mannheim, University of Heidelberg, Mannheim, Germany; 4 Department of Gynecology and Obstetrics, University Medical Center Mannheim, University of Heidelberg, Heidelberg, Germany; 5 Department of Child and Adolescent Psychiatry and Psychotherapy, Central Institute of Mental Health, Medical Faculty Mannheim, University of Heidelberg, Germany and Department of Psychology, University of Potsdam, Potsdam, Germany

We welcome the ongoing interest in our work^1^ and the opportunity to address^2^ concerns with respect to: (i) controlling for sex/ethnicity; (ii) DNA extraction and (iii) telomere length (TL) measurement methodology.

(i) It is well known that sex and ethnicity can affect TL, and both were considered in the analyses and discussed with the reviewers. They were not included in the manuscript for reasons of brevity but we are happy to present them now: including sex in the model does not change the association between prenatal maternal stress and newborn TL (sex: *ß* = −.15, *p* = .009; maternal perceived stress: *ß* = −.13, *p* = .02). Analyses in individuals with Caucasian parents yields results comparable to those obtained for the total sample for newborns (*ß* = −.18, *p* = .003, *n* = 273; total sample *ß* = −.14, *p* = .015) and for mothers (*ß* = −.10, *p* = .096, *n* = 274; total sample *ß* = −.11, *p* = .055). Thus the reported associations are not confounded by sex or ethnicity.
(ii) As explicitly stated in the manuscript, Qiagen kits rather than the standard Chemagen method were used for 14 samples due to a low volume of cord blood. The *t*-test for independent samples was used to determine differences in mean TL resulting from the different extraction methods (*t*_(13.38)_ = 2.45, *p* = .029). This was statistically controlled for in the analyses.
(iii) Contrary to the statement of Esteves et al., quality thresholds for the measurements were presented: one concerning cycle difference between technical duplicates for T and S, and one concerning the linear range of the assay. Our coefficient of variation (CV) was at the lower end of the range reported by other laboratories, and is comparable to that reported in our previous large scale studies.^3^ As outlined in our paper, it is not possible to report an inter-run CV for all samples, as only 128 samples were measured on two occasions. Absolute quantification is not performed against a standard curve. The Rotor-Gene Q comparative quantification software is used to quantify T and S levels relative to K562. Amplification efficiency is calculated and used in the quantification rather than assuming 100% efficiency. Therefore, any minor differences in efficiency between runs are already corrected for within our measurement values. While we minimise the effect of technical variation between batches as much as possible (single reagent batches, consistent equipment and assay QC) our analysis revealed a small batch effect. Repeating the analyses without accounting for batch effects did not change the association between prenatal maternal stress and children’s TL (*ß* = −.13, *p* = .027 compared to *ß* = −.14, *p* = .015 as reported) as well as between maternal lifetime psychiatric disorder and maternal TL (*ß* = −.12, *p* = .028 compared to *ß* = −.11, *p* = .055 as reported) indicating that batch effects are of marginal importance.

We remain confident that our methods and analyses are solid, and naturally share the call of Esteves et al. for larger samples. While our cohort is still the largest to date to show an association between maternal stress during pregnancy and TL in the offspring, larger studies and meta-analyses are needed for a more comprehensive exploration of the multiple factors that impact TL.

## References

[1] Send TS, Gilles M, Codd V, Wolf I, Bardtke S, Streit F, Denniff M, Laucht M, Samani NJ, Deuschle M, Rietschel M, Witt SH (2017). Telomere length in newborns is related to maternal stress during pregnancy. Neuropsychopharmacology.

[2] Send TS, Gilles M, Codd V, Wolf I, Bardtke S, Streit F, Denniff M, Laucht M, Samani NJ, Deuschle M, Rietschel M, Witt SH (2017). Telomere length in newborns is related to maternal stress during pregnancy. Neuropsychopharmacology.

[3] Codd V, Nelson CP, Albrecht E, Mangino M, Deelen J, Buxton JL (2013). Identification of seven loci affecting mean telomere length and their association with disease. Nat Genet.

